# Exogenous amino acids and saccharides mediate the sensitivity of *Klebsiella pneumoniae* phage

**DOI:** 10.1128/spectrum.01902-26

**Published:** 2026-09-23

**Authors:** Jieting Pan, Wanting Shan, Zhu Chen, Mengye Ma, Bingqing Xu, Jiasong Xie, Xibang Liu, Lijian Ding, Liming Jiang

**Affiliations:** 1 Department of Respiratory and Critical Care Medicine, The Affiliated Yangming Hospital of Ningbo University (Yuyao People’s Hospital), Ningbo University 47862, Ningbo, Zhejiang, China; 2 Ningbo Key Laboratory of Malignant Tumor Immunodiagnosis and Immunotherapy, Health Science Center, Ningbo University 74633, Ningbo, Zhejiang, China; 3 School of Basic Medical Sciences, Health Science Center, Ningbo University 74633, Ningbo, Zhejiang, China; 4 Department of Clinical Laboratory Medicine, Ningbo No.2 Hospital, Wenzhou Medical University, Ningbo, Zhejiang, People’s Republic of China; 5 School of Marine Sciences, Ningbo University 631318, Ningbo, Zhejiang, China; 6 School of Pharmacy, Health Science Center, Ningbo University 74633, Ningbo, Zhejiang, China; 7 School of Pharmacy, Changsha Medical University 216793, Changsha, China; University of California San Diego Pathology, La Jolla, California, USA

**Keywords:** *Klebsiella pneumoniae*, phage sensitivity, carbon sources, phage, phage resistance

## Abstract

*Klebsiella pneumoniae* is a major opportunistic pathogen responsible for severe infections, particularly in nosocomial settings. The increasing prevalence of multidrug-resistant strains has greatly limited the effectiveness of conventional antibiotics, highlighting the urgent need for alternative therapeutic strategies. Phage therapy has emerged as a promising approach; however, its efficacy is often influenced by bacterial physiological states, leading to variability in phage susceptibility. Amino acids and saccharides, as essential nutritional components, play important roles in bacterial growth and metabolism. Nevertheless, the mechanisms by which these nutrients affect bacterial physiological states and subsequently modulate susceptibility to phage infection remain poorly understood. In this study, the effects of 18 exogenous amino acids and eight saccharides on the growth of *K. pneumoniae* were assessed, and the variations in phage adsorption sensitivity of the treated bacteria were analyzed. Additionally, the impact of exogenous carbon sources on bacterial characteristics was further investigated through minimum inhibitory concentration (MIC) and biofilm formation assays. Compared with the control group, phage adsorption efficiency was significantly reduced after carbon source treatment. Furthermore, MIC values and biofilm formation capacity were altered to varying degrees, indicating that environmental nutrient conditions may influence the efficacy of phage therapy against drug-resistant *K. pneumoniae* infection.

**IMPORTANCE:**

The success of phage therapy depends not only on phage properties but also on the physiological state of the bacterial host. Although nutrients are fundamental determinants of bacterial metabolism and growth, their effects on phage susceptibility remain poorly characterized. Here, we systematically evaluated the influence of multiple amino acids and saccharides on *K. pneumoniae* and demonstrated that nutrient availability can markedly alter bacterial susceptibility to phage adsorption. In addition, nutrient-induced changes were associated with variations in antimicrobial susceptibility and biofilm formation. These findings highlight the importance of environmental nutritional conditions in shaping phage-host interactions and suggest that nutrient availability should be considered when optimizing phage-based strategies against multidrug-resistant bacterial infections.

## INTRODUCTION

Drug-resistant bacterial infection, especially *K. pneumoniae* infection resistant to carbapenems, has become an important challenge in the field of global public health (1). In particular, *K. pneumoniae* is a kind of Gram-negative *Enterobacter*, which mainly colonizes the upper respiratory tract and intestine of human beings (2, 3). Moreover, it is a major pathogen responsible for human respiratory infections and an important opportunistic pathogen associated with nosocomial infections. The highly virulent strains (hvKP) and multidrug-resistant strains (MDR-Kp) pose a serious threat to clinical treatment (4). The prevalence of carbapenem-resistant *K. pneumoniae* is not only associated with the production of carbapenemases (such as KPC) (5) but may also be closely related to bacterial colonization, phenotypic adaptation, and tolerance strategies (6). The use of antibiotics may lead to the spread of multi-drug resistant pathogens, thereby triggering life-threatening infections. There is an urgent need for alternative schemes that selectively replace traditional antibiotics (7). Therefore, phages, as an emerging strategy to combat drug-resistant bacteria, have re-emerged as a promising therapeutic strategy. Phages are viruses that can infect and lyse bacteria, and they represent a promising alternative treatment option for highly toxic and drug-resistant pathogens such as *K. pneumoniae* infections (8, 9). However, although the host range is narrow and the issue of phage resistance still poses challenges. Phages have the advantages of strong lytic ability, good penetration into biofilms, low side effects, and the ability to work synergistically with antibiotics (10). However, not all phages are suitable for therapeutic applications. An ideal therapeutic phage should preferably exhibit a strictly lytic lifestyle rather than a lysogenic cycle, and should lack undesirable genetic elements such as toxin-associated or antibiotic resistance genes. In addition, favorable host range, stability, and efficient antibacterial activity are important characteristics for evaluating phage candidates for clinical application (11).

In recent years, there has been an increasing number of studies both domestically and internationally on the use of phages for treating infections caused by *K. pneumoniae* (9). What deserves special attention is the ESKAPE (*Enterococcus faecalis*, *Staphylococcus aureus*, *K. pneumoniae*, *Acinetobacter baumannii*, *Pseudomonas aeruginosa*, and *Enterobacter*) pathogens, which have extremely strong evasion capabilities and can evade attacks from various antibiotics (12). This might be one of the reasons why the effectiveness of phage therapy in clinical settings and *in vivo* models is still limited. Meanwhile, an increasing number of studies have shown that the metabolic state of bacteria was an important regulatory factor influencing their physiological functions and stress responses (13, 14). Previous research has indicated that exogenous metabolites such as glucose and amino acids can activate glycolysis and the TCA cycle pathways, thereby increasing the cellular energy metabolism level and changing the growth state and antibiotic sensitivity of bacteria (15, 16). In *K. pneumoniae*, metabolomics studies have found that phage treatment can significantly disrupt the amino acid, nucleotide, and carbon metabolism pathways, suggesting that metabolic reprogramming may be involved in its response to phages (17). Additionally, under nutrient-limiting conditions (such as insufficient carbon sources), bacteria typically exhibit decreased ATP levels and increased (p)ppGpp signaling molecules, which are metabolic characteristics closely related to bacteria entering a low metabolism and tolerance state. Based on these mechanisms, metabolic intervention strategies (such as supplementing specific amino acids or carbon sources) have been proposed to regulate bacterial metabolic activity, thereby enhancing their sensitivity to phages or antibiotics (18), demonstrating certain therapeutic translational potential. Bacterial metabolic state is increasingly recognized as a critical determinant of antibiotic susceptibility and stress adaptation. Carbon source availability profoundly influences central metabolic pathways (19), cellular energy production, and redox balance, thereby shaping bacterial physiological states. Previous studies have primarily focused on the role of carbon metabolism in the formation of antibiotic-tolerant persister cells. However, metabolic regulation is not restricted to this specialized subpopulation. Carbon source-dependent metabolic remodeling may broadly affect growth dynamics, stress responses, and antimicrobial tolerance in the overall bacterial population (20). Therefore, understanding the ways in which carbon availability modulates bacterial physiology may provide new insights into mechanisms underlying antibiotic resistance and survival. However, the mechanisms by which exogenous amino acids and saccharides influence bacterial physiological states and modulate phage susceptibility in *K. pneumoniae* remain poorly understood. In particular, whether nutrient-induced metabolic changes affect phenotypic traits and phage–host interactions remains unclear. In this study, we systematically investigated the effects of a range of exogenous amino acids and saccharides on bacterial physiological traits and phage susceptibility. Rather than focusing solely on individual metabolites, we aimed to explore whether nutrient availability represents an important environmental factor regulating bacterial physiology and phage–host interactions (21).

## RESULTS

### Morphology and characteristics of the isolated phage

**Fig 1:**
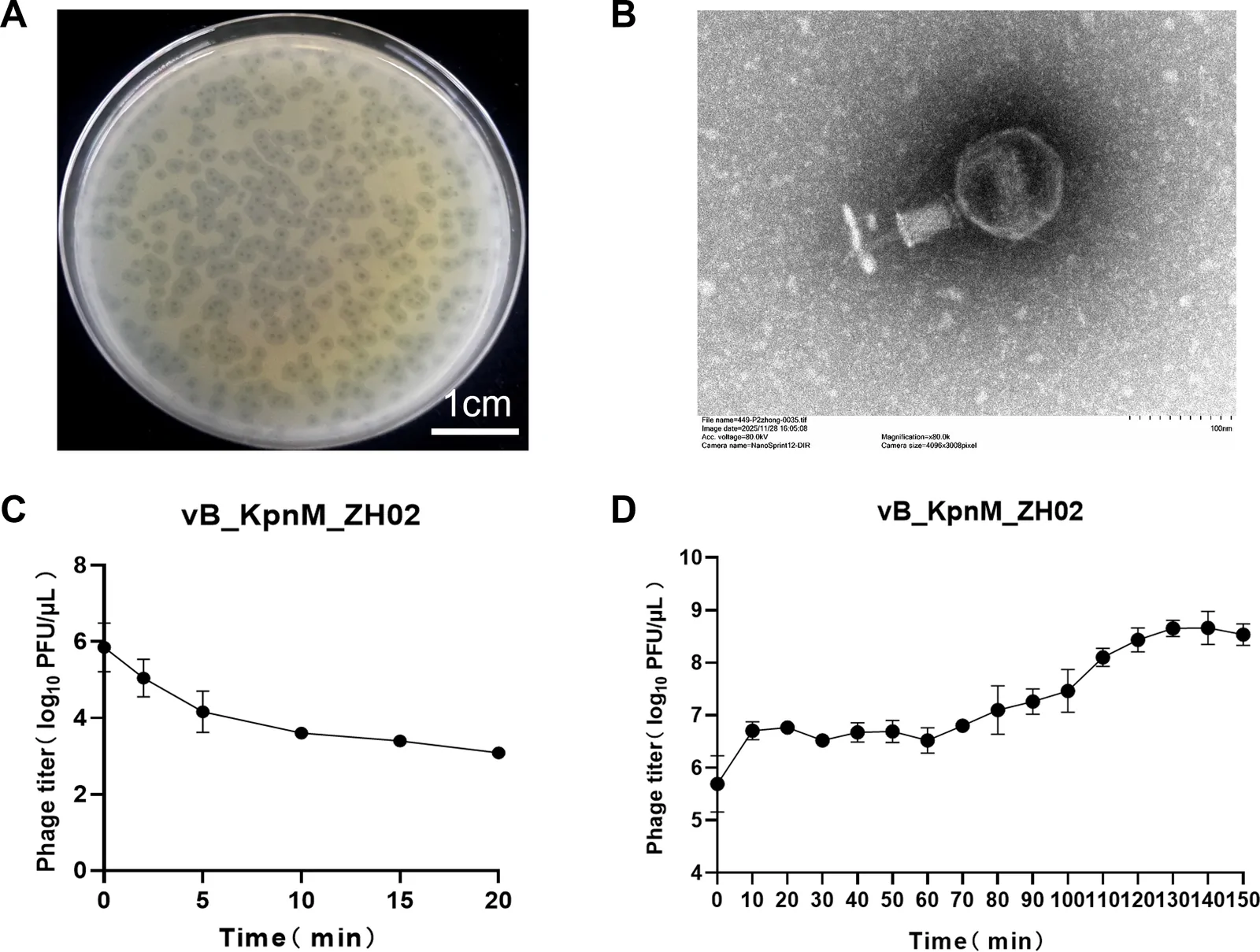
Biological characteristics of phage vB_KpnM_ZH02. (**A**) Plaques formed by vB_KpnM_ZH02 on the host strain *K. pneumoniae* after overnight incubation at 37°C. (**B**) Transmission electron micrograph revealed that vB_KpnM_ZH02 belongs to the family Myoviridae and has a head of 100 nm and a tail of 68 nm. (**C**) Adsorption rate of vB_KpnM_ZH02 inoculated in *K. pneumoniae* culture. (**D**) One-step growth curve of vB_KpnM_ZH02 inoculated in *K. pneumoniae* culture. The values presented are means and standard deviations (SDs) of three independent biological repeats (*n*  =  3).

We isolated a phage vB_KpnM_ZH02 from a river near residential areas in Ningbo, Zhejiang, China, using *K. pneumoniae* as the host bacterium. The phage formed plaques approximately 2 mm in diameter (Fig. 1A). Phage vB_KpnM_ZH02 possessed an icosahedral head with a diameter of approximately 100 nm and a long tail measuring about 68 nm in diameter. Based on the classification criteria established by the International Committee on Taxonomy of Viruses (ICTV), this phage was assigned to the family Myoviridae.

The adsorption kinetics of phage vB_KpnM_ZH02 are shown in Fig. 1C. The titer of free phages decreased rapidly within the first few minutes after infection and dropped markedly at approximately 6 min, indicating efficient adsorption to host cells. Thereafter, the number of unadsorbed phages remained stable, suggesting completion of the adsorption process.

The one-step growth curve revealed a latent period of about 10 min (Fig. 1D), during which the phage titer remained unchanged. A rapid increase in titer was subsequently observed during the lytic phase, reaching a plateau at approximately 110–120 min. The high burst size inferred from the titer increase suggests strong replication efficiency and lytic activity of the phage.

### Influence of temperature, pH, and MOI on phage biological properties

Temperature, pH, and multiplicity of infection (MOI) are important factors affecting phage replication and lytic activity. To evaluate the physicochemical stability of phage vB_KpnM_ZH02, phage suspensions were incubated at different temperatures (4°C–80°C) and pH conditions (pH 3–11), followed by determination of the remaining titers. In addition, the optimal MOI of phage vB_KpnM_ZH02 was determined. All experiments were performed in triplicate, and the results are presented as mean values with deviations indicated in the graphs.

**Fig 2:**
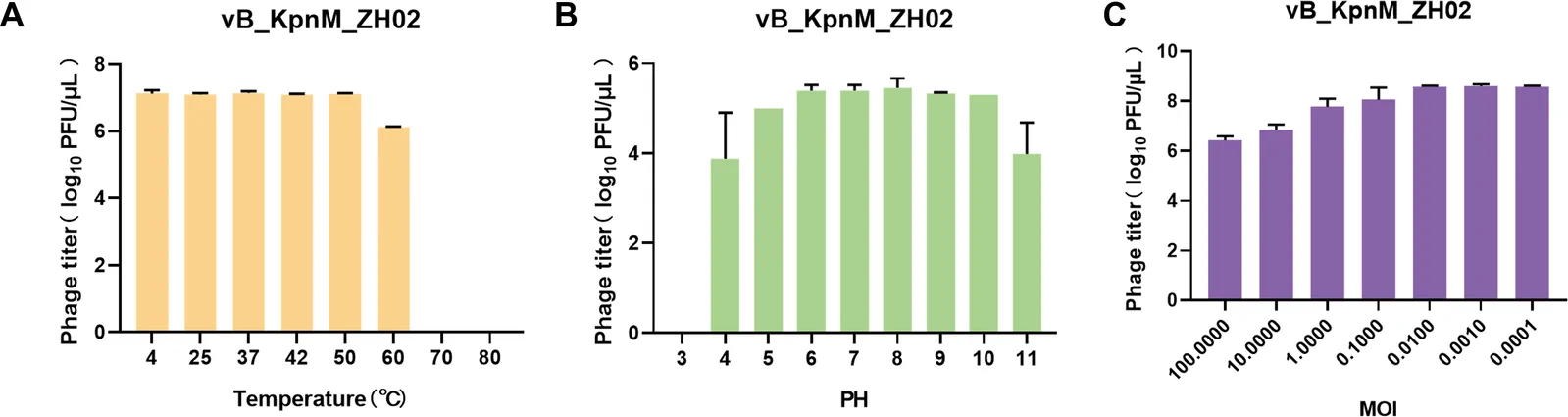
Optimum temperature, pH, and MOI of phage vB_KpnM_ZH02. (**A**) Stability of phage vB_KpnM_ZH02 under various temperature (4°C–80°C) conditions; (**B**) stability of vB_KpnM_ZH02 was tested at different pH values (3–11); (**C**) optimal multiplicity of infection (MOI) determination.

Phage vB_KpnM_ZH02 maintained stable titers between 4°C and 37°C. However, a significant reduction in titer was observed at 60°C, and the phage was completely inactivated at 70°C (Fig. 2A). Phage activity was best preserved within a pH range of 6–8. Strongly acidic conditions (pH 3) led to complete loss of infectivity (Fig. 2B). The phage also demonstrated tolerance to alkaline conditions, with most of the particles remaining viable after 1 h at pH 11, whereas nearly all infectivity was lost after exposure to pH 3 for 1 h.

For optimization of phage amplification, various MOI values were evaluated. Increasing the MOI from 0.1 to 100 resulted in a gradual decrease in phage yield, while the maximum production was achieved at an MOI of 0.01 (Fig. 2C).

### Genomic characteristics of phage

**Fig 3:**
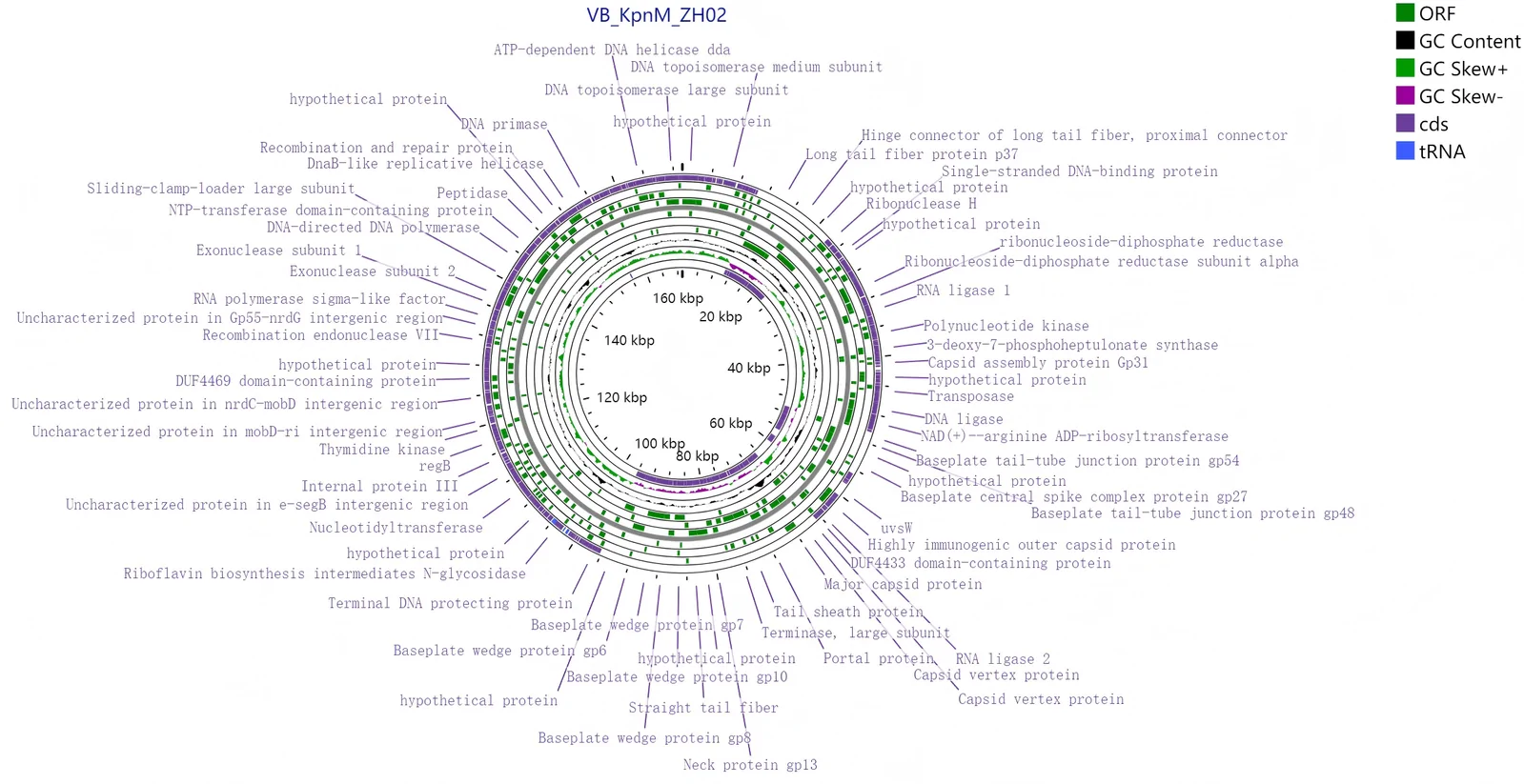
Genomic characterization of bacteriophage vB_KpnM_ZH02. Annotated circular genome map of phage vB_KpnM_ZH02.

VB_KpnM_ZH02 has a linear double-stranded DNA genome with a total length of 169,217 base pairs and a GC content of 47%. The genome contains a total of 276 ORFs, of which 60 are predicted to encode hypothetical proteins, and no tRNA coding region was detected(Fig. 3).

### Phage lytic spectrum

The host range of phage vB_KpnM_ZH02 is shown in Table 1. In this experiment, the differences in phage lysis characteristics were revealed by the analysis of host lysis spectrum. The experimental data showed that vB_KpnP_FU03 and vB_KpnP_DA01 showed narrow-band cracking characteristics, and only their host bacteria could be cracked, which indicated that these two phages had strong host specificity. Phage vB_KpnM_ZH02 has a broad cleavage spectrum, which can not only cleave its host bacteria but also other serotypes of *K. pneumoniae*.

**TABLE 1:** The host range of phage vB_KpnM_ZH02^*a*^

Serial number	Host bacteria name	Host bacteria species	vB_KpnP_FU03	vB_KpnP_DA01	vB_KpnM_ZH02
1	N448	*K. pneumoniae*	−	−	+
2	N449	*K. pneumoniae*	+	+	+
3	N456	*K. pneumoniae*	−	−	+
4	ZR	*K. pneumoniae*	−	−	−
5	KPN	*K. pneumoniae*	−	−	−
6	SG-1	*K. pneumoniae*	−	−	−
7	KPN*-*02	*K. pneumoniae*	−	−	−
8	N1785	*K. pneumoniae*	−	−	−
9	N1787	*K. pneumoniae*	−	−	−
10	N82	*E. coli*	−	−	−
11	N83	*E. coli*	−	−	−
12	N85	*E. coli*	−	−	−
13	N97	*E. coli*	−	−	−
14	ECLO-01	*E. cloacae*	−	−	−
15	PA-01	*P. aeruginosa*	−	−	−
16	RIMD	*V. parahaemolyticus*	−	−	−
17	KPN*-*03	*K. pneumoniae*	−	−	−
18	KPN*-*04	*K. pneumoniae*	−	−	−

^*a*^ "+” means there is plaque, and "−"means there is no plaque. The experiment was performed in triplicate.

To quantitatively evaluate the infectivity of phage vB_KpnM_ZH02 against different hosts, the efficiency of plating (EOP) was determined using the original isolation host as the reference strain. As shown in Table 2, compared with the original host strain (EOP = 1), the two additional susceptible strains exhibited lower EOP values and smaller plaques, suggesting a lower efficiency of phage propagation on these hosts compared with the original isolation host.

**TABLE 2:** Efficiency of plating (EOP) of the phage against different *K. pneumoniae* strains

Strain	Host status	PFU (PFU/mL)	EOP
N448	Sensitive strain	1.40 × 10^7^	0.045
N449	Original host	3.68 × 10^8^	1
N456	Sensitive strain	1.63 × 10^7^	0.355

### Influence of amino acids and saccharides on bacterial growth and phenotypic characteristics

**Fig 4:**
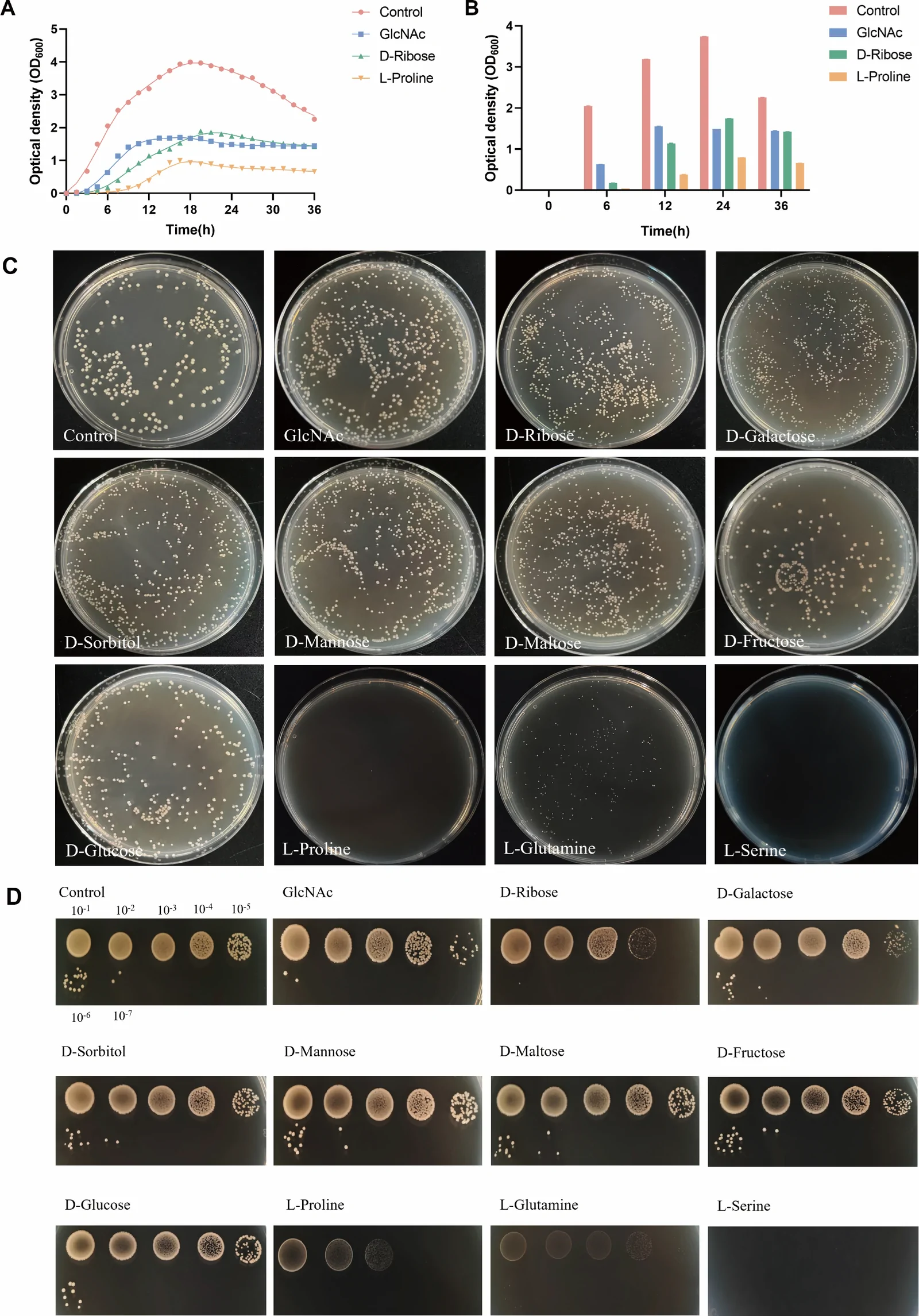
Phenotype of *K. pneumoniae* treated with different carbon sources. (**A**) Growth curve of *K. pneumoniae* treated with different carbon sources. (**B**) Time-dependent changes in OD values of bacterial cultures under different conditions. (**C**) Bacterial cultures were serially diluted (10-fold), and 100 μL of appropriate dilutions was spread onto agar plates for colony counting. (**D**) Bacterial cultures were serially diluted in a 10-fold manner, and 10-μL aliquots of appropriate dilutions were inoculated onto agar plates for colony enumeration.

As shown in Fig. 4, the growth dynamics of *K. pneumoniae* varied markedly under different carbon source conditions. Overall, all groups exhibited a typical growth pattern; however, distinct differences were observed in growth kinetics and final biomass.

During the early stage of growth (0–12 h), no significant differences were observed among the groups, suggesting that initial bacterial adaptation to different carbon sources was relatively comparable. As the culture progressed into the exponential phase, divergence between groups became evident. Certain carbon source-treated groups displayed a more rapid increase in OD_600_, indicating enhanced growth kinetics, whereas other groups showed relatively slower growth trends.

At later time points (12–36 h), clear differences in biomass accumulation were observed. Some carbon source conditions resulted in higher OD_600_ values, suggesting a stronger capacity to support bacterial proliferation, while others maintained lower OD levels, indicating limited growth support. In addition, fluctuations in OD values were observed in some groups during the late growth phase, which may reflect dynamic changes in bacterial population density or metabolic status.

Taken together, these results demonstrate that different carbon sources can significantly influence the growth behavior of *K. pneumoniae*, not only in terms of growth rate but also in final biomass accumulation.

To investigate the resuscitation potential of *K. pneumoniae* under defined nutrient conditions, 18 amino acids and eight saccharides were individually applied as sole carbon sources to induce bacterial recovery. The results showed that *K. pneumoniae* exhibited differential resuscitation patterns on M9 agar supplemented with various carbon sources. Within 24 h, bacterial resuscitation was observed in the presence of GlcNAc, D-sorbitol, D-mannose, D-maltose, and D-glucose. At 36 h, resuscitation became evident under D-fructose, L-glutamine, and L-proline conditions, whereas delayed and slower resuscitation was observed on D-ribose-supplemented plates at 48 h.

Colony morphology appeared largely comparable across polysaccharide-supplemented plates. In contrast, notable differences were observed among amino acid conditions, with significantly fewer colonies formed on L-glutamine plates compared with L-proline plates.

### Antibiotic sensitivity test

Supplementation, the minimum inhibitory concentrations (MICs) of 16 commonly used antibiotics against a clinical isolate of *K. pneumoniae* were determined, using the VITEK 2 Compact automated identification system. The results are summarized in Table 3. The isolates exhibited varying levels of resistance to all 16 antibiotics tested.

**TABLE 3:** Antibiotic sensitivity of *K. pneumoniae* clinical isolates^*a*^

Antibiotic	Abbreviation	Antimicrobial susceptibility testing criteria (mg/mL)		MIC	Interpretive criteria
		S (Susceptible)	R (Resistant)		
Amikacin	AMK	16	64	≥64	R
Ampicillin	‌AMP	8	32	≥32	R
Aztreonam	ATM	4	16	≥64	R
Cefazolin	CFZ	2	8	≥64	R
Cefepime	FEP	2	16	≥64	R
Cefotetan	CTT	16	64	≥64	R
Ceftazidime	CAZ	4	16	≥64	R
Ceftriaxone	CRO	1	4	≥64	R
Ciprofloxacin	CIP	0.25	1	≥4	R
Ertapenem	ETP	0.5	2	≥8	R
Gentamicin	GEN	4	16	≥16	R
Imipenem	IPM	1	4	≥16	R
Levofloxacin	LVX	0.5	2	≥8	R
Nitrofurantoin	NIT	32	128	≥512	R
Piperacillin	PIP	8	32	≥128	R
Trimethoprim–sulfamethoxazole	SXT	2/38	4/76	≥320	R

^*a*^ Interpretation was performed according to CLSI 2023 breakpoints. S, susceptible; I, intermediate; R, resistant.

Subsequently, the MICs of nine selected antibiotics were further evaluated in the laboratory using the *K. pneumoniae* KPN strain. The strain exhibited extensive resistance to multiple antibiotic classes, including carbapenems, aminoglycosides, fluoroquinolones, tetracyclines, and chloramphenicol, indicating a multidrug-resistant (MDR) phenotype with carbapenem resistance characteristics. Based on the antimicrobial susceptibility profile interpreted according to CLSI guidelines, the isolate was identified as CRKP and classified as MDR (Table 4).

**TABLE 4:** Determination of minimum inhibitory concentration (MIC) of nine different antibiotics against *K. pneumonia^a^*

Antibiotic	MIC (mg/mL)
Imipenem	>2,048
Streptomycin	64
Chloramphenicol	512
Amikacin	>2,048
Kanamycin	>2,048
Tetracycline	>2,048
Ampicillin	>2,048
Levofloxacin	1,024

^*a*^ Determination of minimum inhibitory concentration (MIC) of 10 kinds of carbon source-induced bacteria.

Taken together, the antimicrobial susceptibility profiles under different amino acid and saccharide supplementation conditions showed a generally stable resistance phenotype with limited quantitative variations in MIC values. Across all tested metabolic conditions, the MICs of imipenem, amikacin, kanamycin, and ampicillin consistently remained >2,048 µg/mL, indicating persistent high-level resistance to carbapenems and aminoglycosides that was not influenced by the type of carbon or nitrogen source (Table 5).

**TABLE 5:** Determination of minimal inhibitory concentration (MIC) of amino acid-induced 449

Antibiotic	MICs (mg/mL)	
	L-Proline	L-Glutamine
Imipenem	>2,048	>2,048
Streptomycin	512	64
Chloramphenicol	128	256
Amikacin	>2,048	>2,048
Kanamycin	>2,048	>2,048
Tetracycline	64	32
Ampicillin	>2,048	>2,048
Levofloxacin	64	64

For streptomycin, moderate fluctuations were observed. Under amino acid oline) to 64 μg/mL (L-glutamine), whereas under different saccharide conditions, the MIC ranged from 256 to 2048 μg/mL, with higher values detected in certain sugar environments. Chloramphenicol showed relatively stable high MIC values (128–256 μg/mL under amino acid supplementation and 1,024–2,048 μg/mL under saccharide supplementation), suggesting sustained resistance with minor variability. Tetracycline exhibited MICs of 32–64 μg/mL under amino acid treatment and 128–256 μg/mL under saccharide conditions, indicating a slight increase in resistance magnitude in the presence of certain sugars. Similarly, levofloxacin MIC values remained relatively stable (64 μg/mL under amino acid conditions and 256–512 μg/mL under saccharide supplementation) without altering the overall resistance classification (Table 6).

**TABLE 6:** Determination of minimum inhibitory concentration (MIC) of polysaccharide-induced 449

Antibiotic	MICs (mg/mL)							
	N-D	D-Ribose	D-Galactose	D-Sorbitol	D-Sorbitol	D-Sorbitol	D-Fructose	D-Glucose
Imipenem	>2,048	>2,048	>2,048	>2,048	>2,048	>2,048	>2,048	>2,048
Streptomycin	512	256	512	1,024	2,048	1,024	1,024	1,024
Chloramphenicol	1,024	1,024	1,024	1,024	1,024	1,024	1,024	2,048
Amikacin	>2,048	>2,048	>2,048	>2,048	>2,048	>2,048	>2,048	>2,048
Kanamycin	>2,048	>2,048	>2,048	>2,048	>2,048	>2,048	>2,048	>2,048
Tetracycline	128	128	128	128	128	128	256	256
Ampicillin	>2,048	>2,048	>2,048	>2,048	>2,048	>2,048	>2,048	>2,048
Levofloxacin	256	256	256	256	256	256	256	512

Overall, although some antibiotics displayed quantitative MIC variations depending on metabolic conditions, these changes did not modify the strain’s resistance category. The isolate consistently maintained a multidrug-resistant phenotype across all tested nutritional environments.

### Biofilm formation quantification

**Fig 5:**
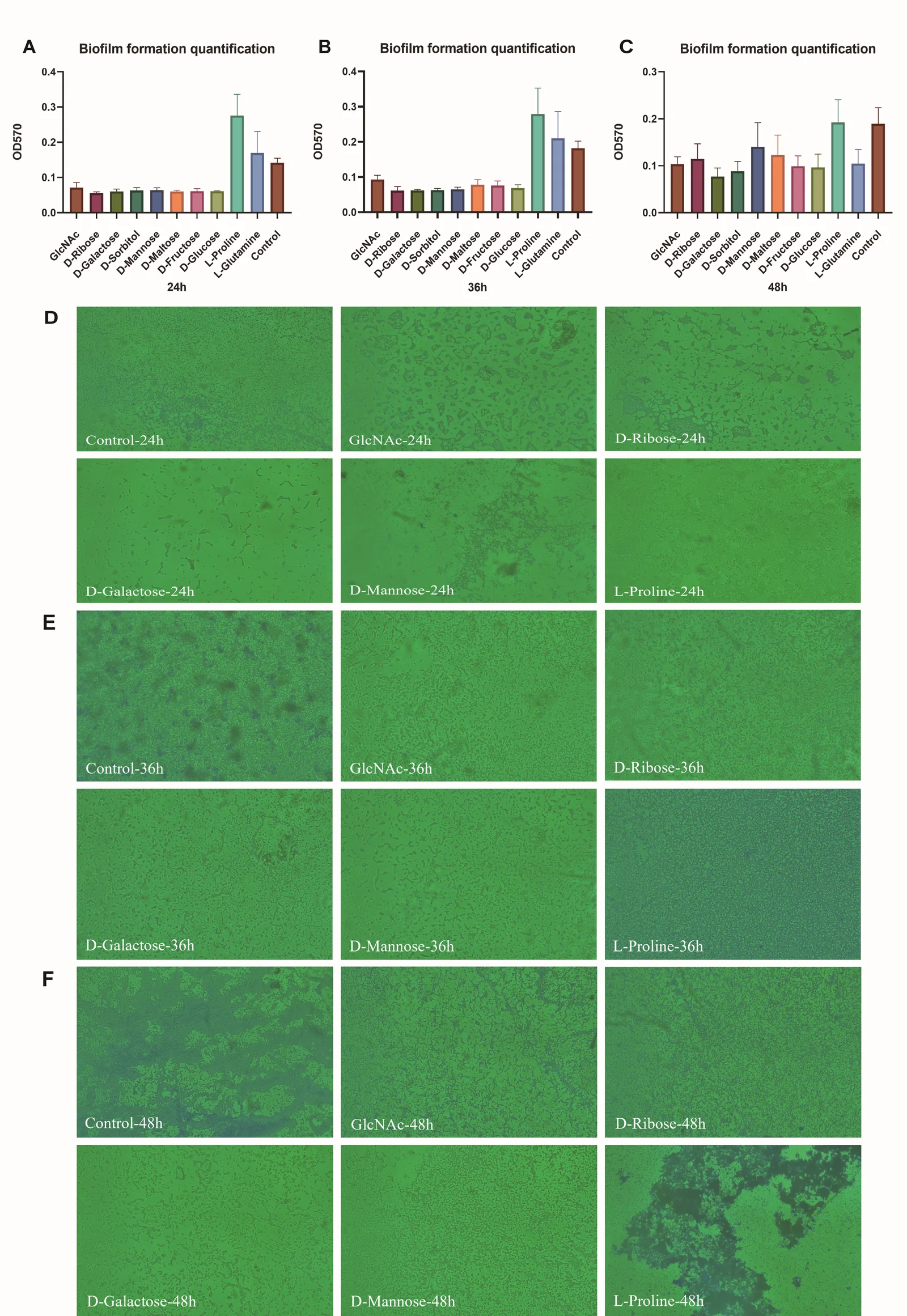
Difference of bacterial biofilm treated with different carbon sources. (**A**) Biofilm formation of bacteria treated with different carbon sources at 24 h. (**B**) Biofilm formation of bacteria treated with different carbon sources at 36 h. (**C**) Biofilm formation of bacteria treated with different carbon sources at 48 h. (**D-F**) Microscopic observation of biofilm formation by *K. pneumoniae* at 24 h (**D**), 36 h (**E**), and 48 h (**F**) following treatment with different carbon sources.

To evaluate whether exposure to exogenous amino acids and saccharides affects the biofilm-forming ability of *K. pneumoniae* relative to the untreated control, biofilm biomass was measured at 24, 36, and 48 h (Fig. 5). The biofilm biomass remained relatively stable between 24 and 36 h, with a significant increase observed at 48 h. The highest level of biofilm formation was recorded under the L-proline condition, while other conditions exhibited comparatively lower biofilm formation capacities. These results indicate that the tested conditions have a significant impact on the biofilm formation potential of the bacteria over time.

The enhanced biofilm formation under L-proline supplementation may be associated with metabolic activation and increased production of extracellular polymeric substances (EPS), which are essential for biofilm maturation and structural stability (22). In contrast, the relatively lower biofilm biomass observed under other conditions suggests that different carbon sources may differentially regulate biofilm-associated pathways.

These results indicate that exogenous nutrient conditions play a critical role in modulating biofilm formation dynamics over time, which may further influence bacterial physiological states and potentially affect susceptibility to phage infection (23).

### Determination of intracellular ATP content

**Fig 6:**
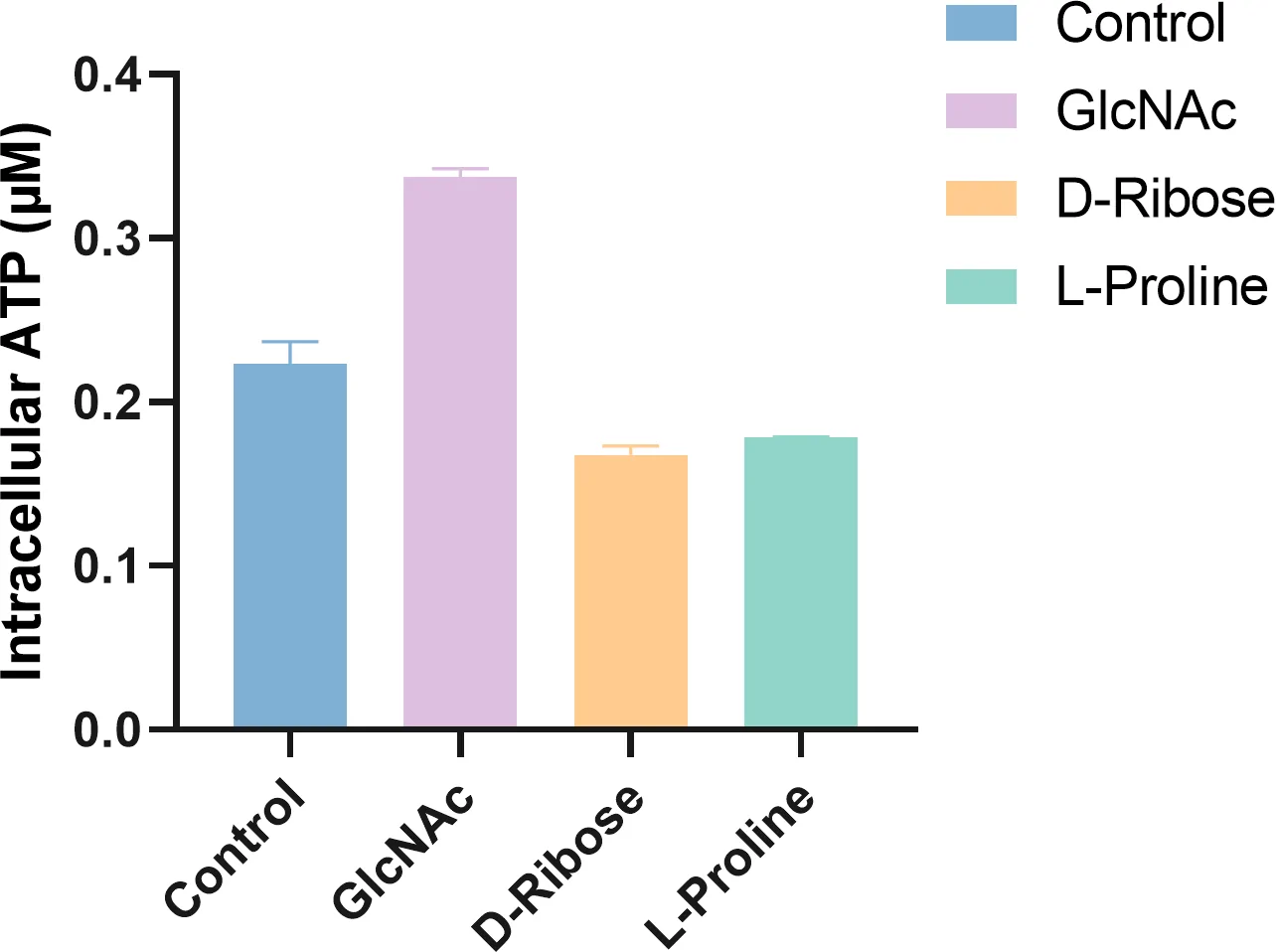
Intracellular ATP quantification. The ATP levels of *K. pneumoniae* and bacteria treated with different carbon sources were measured at OD_600_ = 0.5 by ATP content determination kit.

To measure the intracellular ATP levels of different bacteria at the same optical density, we used an ATP detection kit to assess ATP content in *K. pneumoniae* and *K. pneumoniae* treated with different carbon sources, all adjusted to an OD_600_ of 0.5. Compared with the untreated control group, ATP levels were lower in bacteria treated with D-ribose and L-proline, whereas higher ATP content was observed in the GlcNAc-treated group (Fig. 6).

### Amino acids and saccharides modulate phage adsorption

Phage treatment showed variable inhibitory effects on different *K. pneumoniae* strains during the 2–8-h incubation period. Overall, the OD_600_ values of phage-treated groups were lower than those of untreated controls, indicating suppression of bacterial growth. The inhibitory effects differed among carbon source conditions, with some groups showing transient inhibition followed by partial regrowth, whereas the D-galactose-treated group maintained relatively low OD_600_ values throughout the incubation period. In contrast, L-proline-treated bacteria showed limited growth inhibition after phage addition.

**Fig 7:**
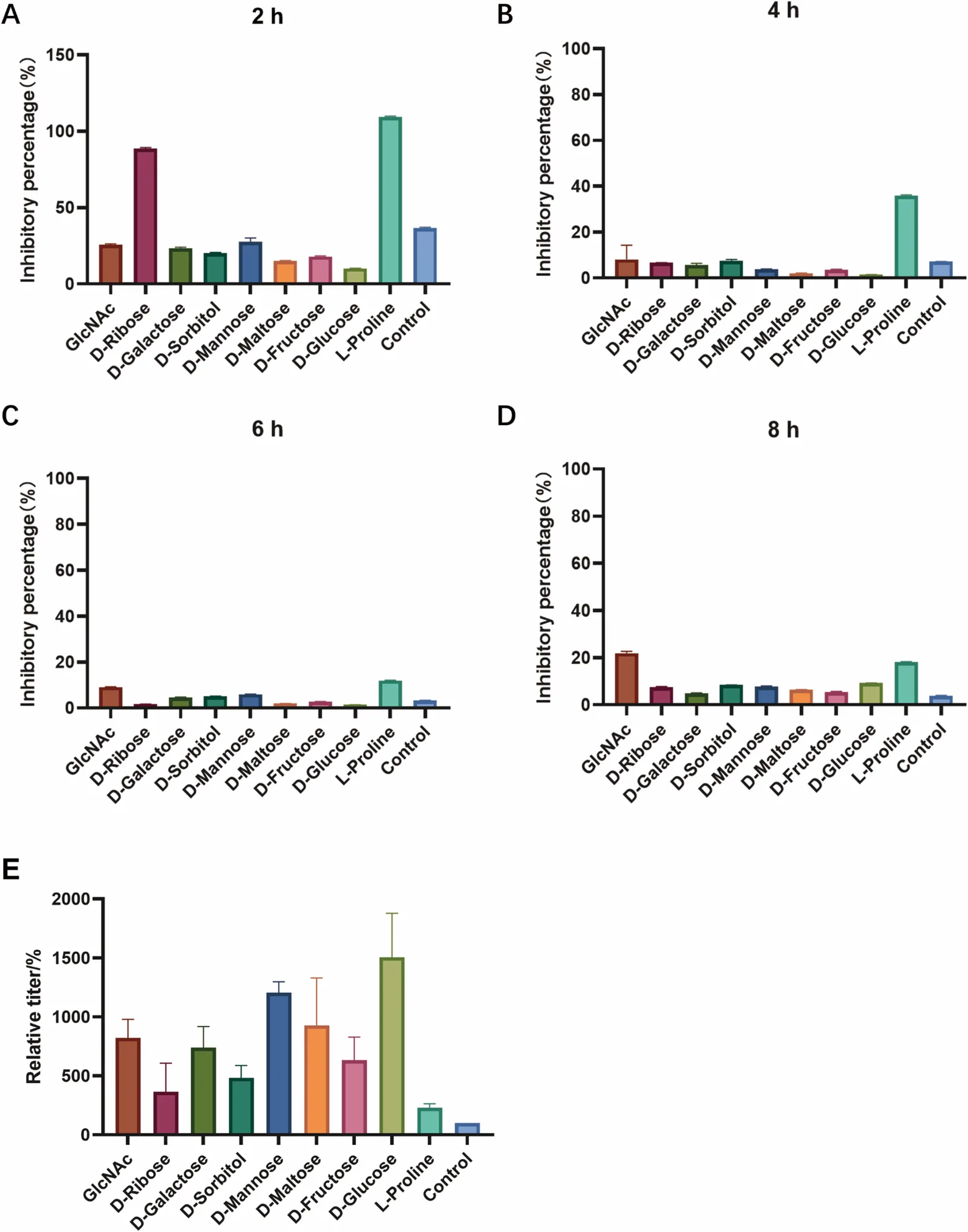
Bacteriostasis percentage of phage to bacteria treated with carbon sources. (**A**) The bacteriostasis percentage of phage at 2 h. (**B**) The bacteriostasis percentage of phage at 4 h. (**C**) The bacteriostasis percentage of phage at 6 h. (**D**) The bacteriostasis percentage of phage at 8 h. (**E**) Phage adsorption efficiency of carbon-treated *K. pneumoniae*.

To determine whether carbon sources affected phage infection efficiency, adsorption assays were performed. Carbon source supplementation significantly reduced phage adsorption efficiency compared with the control group, with the greatest reduction observed in the D-glucose-treated group (Fig. 7). Consistently, EOP analysis showed that all carbon source-treated groups exhibited lower EOP values than the LB control (EOP = 1), indicating reduced productive phage infection under altered nutritional conditions.

Differences in plaque morphology were also observed among carbon source conditions. Plaques formed in the GlcNAc and D-glucose groups were comparable to those in the control group, whereas smaller plaques were observed in the D-ribose and L-proline groups, suggesting that carbon availability may influence phage propagation efficiency (Table 7).

**TABLE 7:** Effect of carbon sources on the efficiency of plating of phage vB_KpnM_ZH02

Carbon source	PFU (PFU/mL)	EOP
LB (control)	3.53 × 10^8^	1
GlcNAc	1.43 × 10^8^	0.405
D-Ribose	6.18 × 10^7^	0.175
D-Glucose	5.4 × 10^7^	0.153
L-Proline	2.25 × 10^7^	0.064

## DISCUSSION

In this study, we systematically evaluated the effects of exogenous amino acids and saccharides on the physiological characteristics of *K. pneumoniae* and its susceptibility to phage adsorption. Our results showed that different carbon sources exerted distinct influences on bacterial recovery and phage adsorption efficiency. Notably, bacteria exposed to carbon source treatment exhibited reduced phage adsorption efficiency compared with the control group. These findings indicate that nutrient availability plays an important role in shaping bacterial physiological states and modulating phage–host interactions.

Carbon source availability is a critical determinant of bacterial metabolic activity and cellular physiology. Amino acids and saccharides can be funneled into central metabolic pathways, such as glycolysis and the tricarboxylic acid (TCA) cycle, thereby affecting ATP production, redox balance, and biosynthetic fluxes. Previous studies have demonstrated that bacterial metabolic state is closely associated with susceptibility to external stresses, particularly antibiotics (19, 24). It has also been suggested that metabolic activity may influence phage infection processes, as phage replication generally depends on host cellular activity. In particular, metabolically active cells are generally more permissive to phage replication, whereas dormant or low-energy states are associated with reduced phage sensitivity (25). Therefore, it is reasonable to speculate that the observed differences in bacterial recovery and phage susceptibility under various carbon sources may be associated with nutrient-driven metabolic remodeling. Such metabolic changes may influence bacterial physiological states, including growth activity, cellular energy availability, and surface-associated properties, thereby affecting different stages of phage infection (26).

Phage adsorption is the initial and essential step of phage infection and is primarily determined by the presence, accessibility, and structural characteristics of specific receptors on the bacterial surface. These receptors typically include lipopolysaccharides (LPS), capsule polysaccharides, and outer membrane proteins (27). In *K. pneumoniae*, capsule polysaccharides are important determinants of phage adsorption and specificity, as certain phages recognize and degrade capsule structures via depolymerases. Nutrient conditions have been reported to influence the synthesis and composition of these surface structures, particularly capsule polysaccharides and membrane-associated components. In *K. pneumoniae*, capsule production is closely linked to environmental conditions and metabolic status, and plays a critical role in bacterial virulence and surface architecture (28). In addition, recent studies have highlighted that metabolic regulation can broadly influence virulence-associated phenotypes in *K. pneumoniae*, further supporting the link between nutrient availability and bacterial physiological remodeling (29). Therefore, it is possible that the altered phage adsorption observed under specific carbon source conditions may be attributed to nutrient-induced alterations in receptor expression or increased accessibility of binding sites. Therefore, it is possible that the enhanced phage adsorption observed under specific carbon source conditions may be attributed to nutrient-induced alterations in receptor expression or increased accessibility of binding sites. However, the precise molecular mechanisms underlying these changes remain unclear and require further investigation. In addition to adsorption-related barriers, bacteria have evolved multiple defense mechanisms that interfere with subsequent stages of phage infection. These include restriction–modification systems, CRISPR-Cas immunity, abortive infection systems, and other antiviral defense pathways, which can prevent phage genome replication and progeny production (30, 31). Importantly, bacterial physiological states may influence these defense processes by regulating cellular energy metabolism, stress responses, and resource availability (20). Therefore, the altered phage susceptibility observed under different carbon source conditions may reflect nutrient-induced changes in host physiology that affect both early adsorption events and subsequent intracellular infection processes. Further studies are required to identify the specific molecular pathways involved.

In addition to phage adsorption, exogenous carbon sources may also influence other physiological traits, such as biofilm formation and antibiotic susceptibility. Biofilm formation is tightly associated with nutrient availability and metabolic status, as it involves coordinated regulation of extracellular polymeric substances and quorum sensing pathways (32). Similarly, bacterial metabolism has been shown to modulate antibiotic susceptibility by affecting energy-dependent processes, including drug uptake, efflux activity, and stress responses (19). Therefore, the phenotypic variations observed in this study likely reflect a broader metabolic remodeling process induced by different carbon sources, which simultaneously impacts multiple aspects of bacterial physiology.

Taken together, these findings highlight the important role of nutrient availability in modulating the physiological state of *K. pneumoniae*, particularly in terms of metabolic activity, surface properties, and susceptibility to phage infection. By influencing bacterial metabolic pathways and potentially altering surface-associated structures, exogenous carbon sources may indirectly regulate phage–host interactions.

Although the present study primarily provides phenotypic evidence, the results suggest a potential link between nutrient-induced metabolic reprogramming and changes in phage adsorption efficiency, as bacterial metabolic state has been reported to influence susceptibility to external stresses (24). Further investigations, such as transcriptomic analysis and characterization of phage receptors, will be necessary to elucidate the underlying molecular mechanisms. In addition, exploring how specific metabolic pathways contribute to variations in phage susceptibility may provide new insights into optimizing phage-based strategies against multidrug-resistant *K. pneumoniae*.

## MATERIALS AND METHODS

### Bacterial strains and growth conditions

The bacterial strain used in this experiment was isolated from clinical samples of *K. pneumoniae*. This strain was chosen to represent the clinical strains of *K. pneumoniae*, commonly associated with infections in hospital settings. LB broth was prepared using tryptone, yeast extract, and NaCl at final concentrations of 10, 5, and 10 g/L, respectively. The strain was cultured at 37°C with shaking at 240 rpm. LB soft agar containing 0.5% (w/v) agar was used for phage plaque confirmation.

M9 minimal medium was prepared by mixing 10 mL of 0.1 M CaCl_2_, 10 mL of 0.1 M MgSO_4_, and 840 mL of ddH_2_O, and a sterilized 10× salt solution containing 20 g/L NaCl, 30 g/L KH_2_PO_4_, 70 g/L Na_2_HPO_4_, and 10 g/L NH_4_Cl was prepared in ddH_2_O. The saccharide concentration was set to 0.4%, used for resuspending *K. pneumoniae* cells. The amino acids and their respective concentrations used to resuspend *K. pneumoniae* cells are provided in Table 8 (33). The amino acids and saccharides used in this study were purchased from Aladdin, unless otherwise specified.

**TABLE 8:** Levels of amino acids in M9 minimal medium

Amino acid	Final concentration (μg/mL)	Stock	Amount per liter
L-Alanine	375	5%	7.5 mL
L-Arginine	725	10%	7.25 mL
L-Aspartic acid	375	5%	7.5 mL
L-Cysteine	250	10%	2.5 mL
L-Histidine	210	10%	2.1 mL
L-Glutamic acid	375	5%	7.5 mL
L-Glutamine	375	5%	7.5 mL
L-Glycine	550	10%	5.5 mL
L-Isoleucine	210	5%	4.2 mL
L-Leucine	205	5%	4.1 mL
L-Lysine	375	5%	7.5 mL
L-Methionine	125	10%	1.25 mL
L-Phenylalanine	375	5%	7.5 mL
L-Proline	820	20%	4.1 mL
L-Serine	210	10%	2.1 mL
L-Threonine	410	10%	4.1 mL
L-Tryptophan	90	1.25%	7.1 mL
L-Valine	210	5%	4.2 mL

### *K. pneumoniae* cells revived on amino acids and saccharides

The amino acids used for cell resuscitation were selected according to previously reported methods (33). Eighteen amino acids, including L-alanine, L-arginine, L-aspartic acid, L-cysteine, L-histidine, L-glutamic acid, L-glutamine, L-glycine, L-isoleucine, L-leucine, L-lysine, L-methionine, L-phenylalanine, L-proline, L-serine, L-threonine, L-tryptophan, and L-valine, were evaluated. Briefly, serially diluted *K. pneumoniae* cells were plated onto M9 minimal agar supplemented with individual amino acids as the sole carbon source and incubated at 37°C for 96 h. In addition, eight saccharides, including N-acetyl-D-glucosamine (GlcNAc), D-ribose, D-galactose, D-sorbitol, D-mannose, D-maltose, D-fructose, and D-glucose, were assessed using the same procedure, with incubation at 37°C for 48 h.

### Bacterial growth curve analysis

Bacterial strains in the experimental groups were cultured in M9 medium supplemented with individual carbon sources (GlcNAc, D-ribose, or L-proline), while the untreated control group was cultured in LB medium. All cultures were incubated overnight at 37°C with shaking at 240 rpm. The cultures were then diluted to an initial OD_600_ of 0.5 and re-inoculated into the corresponding fresh media. The cultures were subsequently incubated at 37°C with shaking at 240 rpm for 36 h. Samples were collected every 1.5 h, and bacterial growth was monitored by measuring OD_600_ using a spectrophotometer. All experiments were performed in triplicate.

### Phage isolation and purification

Phages were isolated from residential sewage sump samples collected in Ningbo, Zhejiang, China. Phage isolation was performed as previously described, with minor modifications (34). Briefly, samples were suspended in phosphate-buffered saline, shaken, centrifuged, and filtered through a 0.22-μm membrane. The filtrates were enriched with exponentially growing *K. pneumoniae* in LB broth for 24 h and subsequently filtered. Phages were detected by the double-layer agar method, in which serially diluted filtrates were mixed with host bacteria in molten LB soft agar and overlaid onto LB agar plates. After incubation, individual plaques were picked and purified by three successive rounds of plaque purification.

### Adsorption rate and growth curve

The adsorption kinetics of phage vB_KpnM_ZH02 were evaluated by incubating phage suspension (1 × 10⁸ PFU/mL) with logarithmic-phase *K. pneumoniae* at an MOI of 0.1 and 37°C. Samples were collected at different time points (0–30 min), and phage titers were determined to calculate the proportion of unadsorbed phages. The one-step growth curve assay was performed, as previously described (35). Briefly, *K. pneumoniae cells* were infected with phage vB_KpnM_ZH02 at an MOI of 0.1 after a 10-min adsorption period. Following removal of unadsorbed phages, infected cells were resuspended in fresh LB medium, and samples were collected at 10-min intervals for 2 h to determine phage titers using the double-layer agar method.

### Effects of temperature, pH, and MOI on phage activity

#### Thermotolerance

The phage vB_KpnM_ZH02 stock was diluted to 1  × 10^8^ PFU/mL^−1^ with LB broth culture. One milliliter of diluted phage was incubated at either 4°C, 25°C, 37°C, 42°C, 50°C, 60°C, 70°C, and 80°C for 1 h. The phage titer was determined at different temperatures. The experiment was repeated three times (36).

#### Optimum pH

To evaluate phage stability under different pH conditions, 0.99 mL of buffers with pH values ranging from 3 to 11 were dispensed into sterile 1.5 mL microcentrifuge tubes. The buffers included citrate buffer (50 mmol L⁻¹; pH 3–5), phosphate buffer (50 mmol L⁻¹; pH 6-8), Tris-HCl buffer (50 mmol L⁻¹; pH 9), and sodium carbonate buffer (50 mmol L⁻¹; pH 10–11). Sodium citrate, Na2HPO4, Tris-HCl, and sodium carbonate were used for buffer preparation. Phage vB_KpnM_ZH02 was added to each tube to achieve a final concentration of 1 × 10⁸ PFU mL⁻¹. The mixtures were incubated at 37°C for 1 h, after which phage titers were determined. All experiments were performed in triplicate.

#### Optimum MOI

The multiplicity of infection (MOI) is the ratio of phage to host bacteria during the initial infection. Phage vB_KpnM_ZH02 stocks were added to *K. pneumoniae* culture medium according to the MOIs of 0.0001, 0.001, 0.01, 0.1, 1, 10, and 100, then cultured at 37°C for 12 h. The cultures were centrifuged at 6,000 × *g* for 5 min at 4°C; the supernatant was filtered with a 0.22-µm filter, and the titer of each phage solution was determined through the double plate method. The experiment was repeated three times (37).

### Transmission electron microscopy

The morphology of phage vB_KpnM_ZH02 was examined by transmission electron microscopy (TEM). Phage suspensions (approximately 4 × 10⁸ to 4 × 10⁹ PFU/mL) were applied onto copper grids coated with carbon-supported formvar films, followed by negative staining with 2% (w/v) uranyl acetate (pH 4). The prepared samples were observed using a HITACHI TH-7800 transmission electron microscope at an accelerating voltage of 80 kV at Ningbo University (Ningbo, China). Taxonomic classification of phage vB_KpnM_ZH02 was performed according to the criteria established by the International Committee on Taxonomy of Viruses (38).

### Sequencing and analysis

Phage genomic DNA was extracted from purified phage lysates using a commercial DNA extraction kit according to the manufacturer’s instructions. Prior to extraction, the phage lysate was treated with DNase I and RNase A at 37°C for 90 min to remove host nucleic acids, followed by enzyme inactivation at 75°C for 7 min. The DNA was subsequently purified through proteinase K digestion and column-based purification steps. Whole-genome sequencing was performed using the Illumina NovaSeq 6000 platform with the TruSeq DNA Nano library preparation kit. Raw sequencing data were assembled using SPAdes (v3.15.0). Open reading frames (ORFs) were predicted using Prodigal (v2.6.3) and further annotated by comparison against the NCBI non-redundant (NR) database using BLAST. The circular genome map of the phage was visualized using Proksee.

### Phage lytic spectrum

The host range of phage vB_KpnM_ZH02 was determined by the spot test method (39). We tested the host range of three types of phages against 9 strains of *K. pneumoniae* and nine other bacterial species (*Escherichia coli*, *Enterobacter cloacae*, *P. aeruginosa*, *Vibrio parahaemolyticus*). Briefly, 10 μL of phage suspension (10⁸ PFU/mL) was spotted onto bacterial lawns, and lytic zones were observed after incubation at 37°C for 12 h. The efficiency of plating (EOP) was further determined by comparing the PFU values obtained on different susceptible strains with that of the original isolation host. EOP values were calculated as the ratio of PFU/mL on the tested strain to PFU/mL on the reference host.

### Antibiotic sensitivity test

Antimicrobial susceptibility of clinical *K. pneumoniae* isolates was determined using the VITEK 2 Compact system (bioMérieux) with GN67 cards. MICs for 16 antimicrobial agents were interpreted as susceptible or resistant according to CLSI 2023 guidelines. The minimum inhibitory concentrations (MICs) of nine antimicrobial agents, including imipenem, streptomycin, chloramphenicol, amikacin, kanamycin, tetracycline, ampicillin, and levofloxacin, were determined against *K. pneumoniae*. Antibiotic stock solutions were prepared at a concentration of 50 mg/mL (except levofloxacin, 10 mg/mL), sterilized by filtration through 0.22 μm membrane filters, aliquoted, and stored at −20°C until use.

The MICs of antimicrobial agents were determined using broth microdilution susceptibility method and interpreted according to the Clinical and Laboratory Standards Institute guidelines (40).

### Biofilm formation quantification

Biofilm formation was quantified using a crystal violet staining assay. Briefly, overnight bacterial cultures were diluted 1:100 in the indicated liquid media, and 200 μL aliquots were dispensed into 96-well plates. Plates were incubated at 37°C for 24 h, after which the supernatant was removed, and wells were washed once with 1× PBS (G4202-500mL) to eliminate planktonic cells. Adherent cells were fixed with paraformaldehyde (BL539A, biosharp), washed with PBS, and stained with 0.1% (w/v) crystal violet (71012314, Hushi) for 10 min. Excess stain was removed by washing twice with 1× PBS. Crystal violet retained by the biofilm was solubilized with PBS, and biofilm biomass was determined by measuring absorbance at 570 nm.

### Measurement of intracellular ATP content

Intracellular ATP levels were quantified using a commercially available ATP assay kit (G4309-48T, Servicebio) in accordance with the manufacturer’s instructions. Briefly, untreated *K. pneumoniae* cultures and bacteria exposed to different carbon source treatments were cultivated to mid-logarithmic phase and adjusted to an OD_600_ = 0.5 prior to analysis. Cells were subsequently processed for ATP determination following the kit protocol. For each condition, including blank controls and treatment groups, three independent biological replicates were included to ensure reproducibility.

### Phage killing curve

Overnight cultures of amino acid-treated or saccharide-treated *K. pneumoniae* were diluted in fresh LB medium and cultured at 37°C with shaking until the OD_600_ reached 0.4–0.6. The cultures were then adjusted to an OD_600_ of 0.3. Phage vB_KpnM_ZH02 was added to the bacterial suspensions, while LB medium or heat-inactivated phage was added as controls. The mixtures were incubated at 37°C with shaking, and bacterial growth was monitored by measuring OD_600_ at 0, 2, 4, 6, and 8 h.

### Phage adsorption assay under different nutrient conditions

Bacterial strains were cultured overnight in M9 medium supplemented with different saccharides and amino acid solutions and adjusted to OD_600_ = 0.5. For the experimental group, 2 mL of treated bacterial suspension was mixed with 10 μL of phage lysate. The positive control consisted of 2 mL of strain 449 bacterial suspension with 10-μL phage, while the negative control contained 2 mL M9 medium with 10-μL phage. After incubation at room temperature for 2 min, the mixtures were immediately filtered, and the phage titer in the supernatant was determined.

To further evaluate whether carbon source-dependent physiological changes influence phage infectivity, *K. pneumoniae* 449 cells were cultured in M9 medium supplemented with GlcNAc, D-ribose, D-glucose, or L-proline, with LB medium as the control. Bacterial cultures were adjusted to OD_600_ = 0.5 and infected with serially diluted phage vB_KpnM_ZH02 using the double-layer agar method. After overnight incubation, plaques were counted, and EOP values were calculated as the ratio of PFU obtained under each carbon source condition to that of the LB control. Plaque morphology and diameter were also recorded.

### Statistical analysis

All experiments were independently repeated two or three times. Data processing was initially carried out using the Excel Analysis ToolPak, followed by statistical analyses with GraphPad Prism (GraphPad Software, CA, USA). Differences were considered statistically significant at *P* < 0.05.

## Data Availability

The complete genome information of phage vB_KpnM_ZH02 was submitted to the NCBI database for accession number PZ466139.1.
